# Wearable cuffless blood pressure tracking: when will they be good enough?

**DOI:** 10.1038/s41371-024-00932-3

**Published:** 2024-07-12

**Authors:** Aletta E. Schutte

**Affiliations:** 1 School of Population Health, University of New South Wales, Sydney, NSW Australia; 2 The George Institute for Global Health, Sydney, NSW Australia; 3 Hypertension in Africa Research Team, MRC Unit for Hypertension and Cardiovascular Disease, North-West University, Potchefstroom, South Africa; 4 SAMRC/Wits Developmental Pathways for Health Research Unit, Department of Paediatrics, Faculty of Health Sciences, University of the Witwatersrand, Johannesburg, South Africa

**Keywords:** Hypertension, Diagnosis

## Abstract

Wearable health monitoring is a multibillion-dollar industry. But the holy grail is probably getting it right for blood pressure monitoring without a cuff, because raised blood pressure is very common and the leading cause of death in the world. Many have tried and failed, but industry is persisting: numerous cuffless wearable blood pressure devices are on the market, several technologies have been developed, hundreds of patents are registered every year, and some devices already have regulatory approval. However, to convince the die-hard blood pressure critic is a different ball game. To understand the challenges of currently accepted methods *and* cuffless devices, I performed a 24-h blood pressure monitoring self-test, including measurements when awake, asleep and watching an intense match of the Rugby World Cup final, with the purpose to demonstrate the challenges and opportunities we face. Blood pressure was monitored using five different devices simultaneously: validated left and right arm cuff blood pressure, and three cuffless wearable devices (wrist-band, chest patch and a ring). Whilst none of these devices proved to be perfect in capturing a physiologically challenging measure, namely blood pressure, it emphasised that our current practice of a single blood pressure measurement in clinical practice should be revisited. It further begs the question of when cuffless measurements will be good enough to incorporate in clinical decision-making.

Wearable health monitoring is a multibillion-dollar industry. But the holy grail is probably getting it right for blood pressure monitoring without a cuff, because raised blood pressure is the leading cause of death in the world [1]. Many have tried and failed, but industry is persisting: numerous devices are on the market, several different technologies have been developed, hundreds of patents are registered every year [2], and some devices already have regulatory approval [3]. However, to convince the die-hard blood pressure critic is a different ball game.

Cuffless blood pressure devices rely on a range of different technologies to estimate blood pressure changes, with most using pulse transit time with ECG, or pulse wave analysis combined with proprietary algorithms (and often artificial intelligence)—relying mostly on an initial calibration with a cuff. Other potential technologies include facial video processing or ultrasound, which may not require calibration with a cuff [2]. Technical challenges with devices may include their accuracy, stability of the measurements post calibration, and how machine learning technology is implemented—in other words, how reliant the algorithm is on hemodynamic components and other demographic inputs, such as sex and age [2].

Although authorities such as the American Heart Association [4] and European Society of Hypertension [2] recognise the major potential of cuffless blood pressure monitoring, both have published statements against their use in clinical practice due to accuracy issues, and uncertainty regarding their usefulness in clinical practice. The question is not so much whether a cuffless wearable device can produce a blood pressure comparable to a cuff device—since this has been proven relatively easy to do—but rather whether cuffless wearables can *track* blood pressure when calm, sleeping and excited [5].

At the same time, we have to admit that blood pressure is a very challenging physiological measure. It changes from second-to-second, day-and-night, and during winter-and-summer, when we are calm or excited [6]. Even our ‘gold standard’ method for diagnosing hypertension, cuff-based 24-h blood pressure monitoring, has poor intra-individual reproducibility across two different days [7].

From a clinical perspective it is understandable that for cuffless blood pressure monitoring to be accepted, there needs to be convincing evidence that the device can track pressure in a similar fashion as (imperfect) 24-h cuff-based monitors. This is due to strong evidence that 24-h cuff blood pressure predicts death and cardiovascular events, with nighttime blood pressure being the most potent predictor of outcome [8–10]. If a cuffless device does not track cuff-based blood pressure, how would a clinician feel confident to make treatment decisions? For this reason, the European Society of Hypertension now recommends stringent criteria for cuffless device manufacturers to validate the accuracy of their products in tracking blood pressure [5]. These include procedures including six validation tests that were developed to evaluate different aspects of so-called “intermittent” cuffless devices: (1) a static test similar to validation tests used for cuff-based devices (absolute accuracy in blood pressure measurement); (2) a device position test (whether it captures changes in hydrostatic pressure); (3) medication treatment test (accuracy in tracking blood pressure lowering); (4) awake/asleep test over 24-h (tracking change in blood pressure); (5) exercise test (tracking blood pressure increases); (6) and the recalibration test (stability in cuff calibration blood pressure over time) [5].

From a patient’s perspective, it is understandable that comfort is tops [11]. A wrist-band, patch or ring that does not disturb nighttime sleep or disrupt daily activities, or cause an anxiety response when the cuff inflates [12], would be much easier to wear for months, let alone 24 hours.

Being a blood pressure critic myself, understanding the challenges of currently accepted methods *and* cuffless devices, I performed a 24-h blood pressure monitoring self-test. While not claiming a flawless study by any means (with an *N* = 1), it merely serves to demonstrate the challenges that we face. I monitored my own blood pressure while using five different devices simultaneously: cuff-based ambulatory monitoring (left and right arm) with two validated WatchBP O3 devices (MicroLife, Switzerland) [13], and three wearable cuffless devices: the Aktiia wrist-wearable (Aktiia SA, Switzerland) [14, 15], Biobeat chest patch (Biobeat Technologies, Israel) [16, 17], and the CAR-T ring (Skylabs, South Korea) [18, 19]. At the start of the 24-h period, the Aktiia device was calibrated using its own supplied upper-arm cuff. The Biobeat and CAR-T ring were calibrated according to device specifications using a validated iHealth Feel (iHealthlabs, USA) upper-arm blood pressure cuff. I then evaluated how well all devices tracked my blood pressure while I was awake, asleep and when watching the 2023 Rugby World Cup final on television (early morning in Sydney). Being an avid Springbok rugby fan, I anticipated a substantial blood pressure response. This is likely a good emotional stressor, since in New Zealand, a strong upswing in acute cardiac hospital admissions during Rugby World Cup tournaments were seen when the All Blacks participated [20].

When reviewing the systolic blood pressure results (Fig. 1 Panel A), it is firstly clear that despite the two cuff-based devices inflating at precisely the same second, there were clear points of discrepancy while awake, asleep and during the rugby match. This may have been due to movement, body position or other factors. However, there were no statistical differences between average awake (*p* = 0.35), asleep (*p* = 0.88) or rugby (*p* = 0.35) blood pressure readings between the left and right arm devices. It is also relevant to mention that systolic blood pressure dipped from an average of 113 mmHg when awake, to 91 mmHg when asleep, rising to an average of 123 mmHg when watching the rugby. Since the blood pressure of the two cuff devices was not statistically different, they were combined and averaged for comparison with the three cuffless devices.

**Fig. 1:**
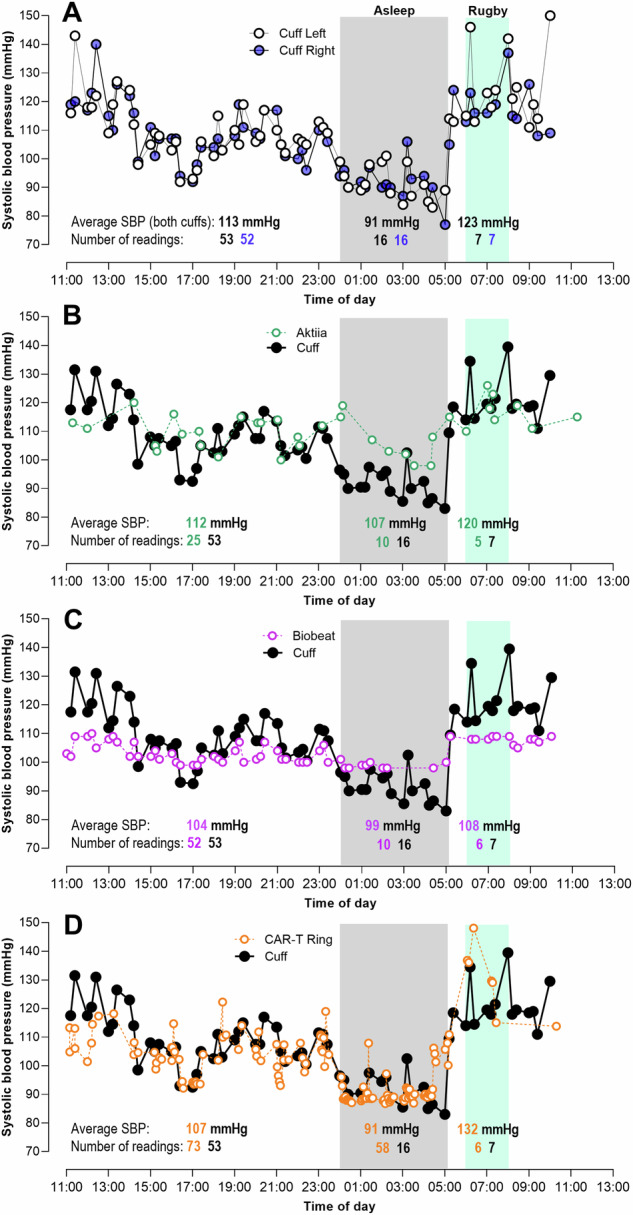
24-H systolic blood pressure, while awake, asleep and while watching the Rugby World Cup Final. **A** Left vs right arm ambulatory cuffs; and average of left and right arm cuff blood pressure vs. **B** Aktiia wrist-worn device; **C** Biobeat chest patch; and **D** CAR-T ring.

The Aktiia device (Fig. 1 Panel B) produced comparable systolic blood pressure when awake (*p* = 0.73) and during the rugby match (*p* = 0.54), but reported higher values when asleep (*p* < 0.001). This finding aligns with our previous independent comparison study in 41 patients [21].

The Biobeat patch device (Fig. 1 Panel C) produced lower systolic blood pressure when awake (*p* < 0.001) and during the rugby match (*p* = 0.005), and higher blood pressure when sleeping (*p* < 0.001), suggesting strong reliance on the original calibration pressure.

The CAR-T ring (Fig. 1 Panel D) showed lower systolic blood pressure when awake (*p* = 0.002), but was comparable when sleeping (*p* = 0.56) and when watching the rugby (*p* = 0.13).

In Fig. 2, similar device comparisons are shown but for heart rate across the 24-h period. Overall, the cuffless devices tracked heart rate quite well compared to the heart rate recorded by the cuff devices.

**Fig. 2:**
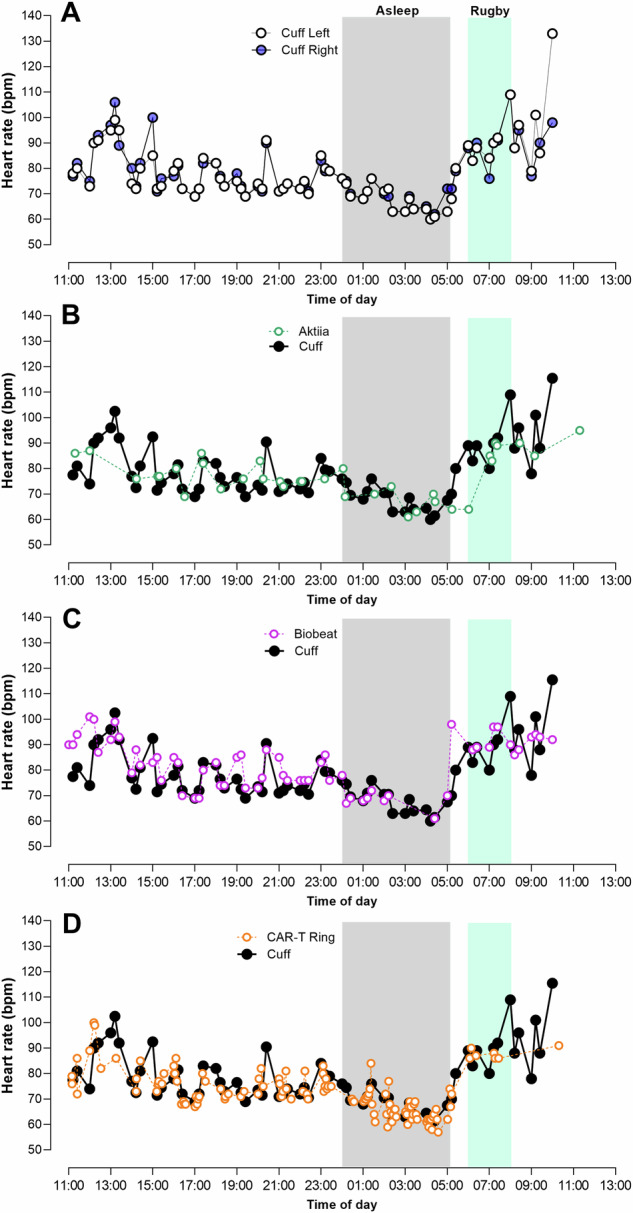
24-H heart rate, while awake, asleep and while watching the Rugby World Cup Final. **A** Left vs right arm ambulatory cuffs; and average of left and right arm cuff heart rate vs. **B** Aktiia wrist-worn device; **C** Biobeat chest patch; and **D** CAR-T ring.

The comparisons above focus only on systolic blood pressure and heart rate, but it is important to also review performance in diastolic blood pressure. In Fig. 3 comparisons are presented in Panel A firstly showing no statistical differences between the left and right cuff-based devices for systolic, diastolic blood pressure and heart rate. In Panel B the differences between the cuffless and cuff-based devices are shown for 24-h, awake, asleep and rugby match recordings, indicating for diastolic blood pressure, mainly overestimation of blood pressure by cuffless devices during the nighttime, but no statistical differences during awake or rugby recordings. Heart rate recordings were comparable between cuff-based devices and the Aktiia and BioBeat devices across the 24-h, but the CAR-T ring underestimated heart rate during the awake period (mean 78 vs 82 bpm).

**Fig. 3:**
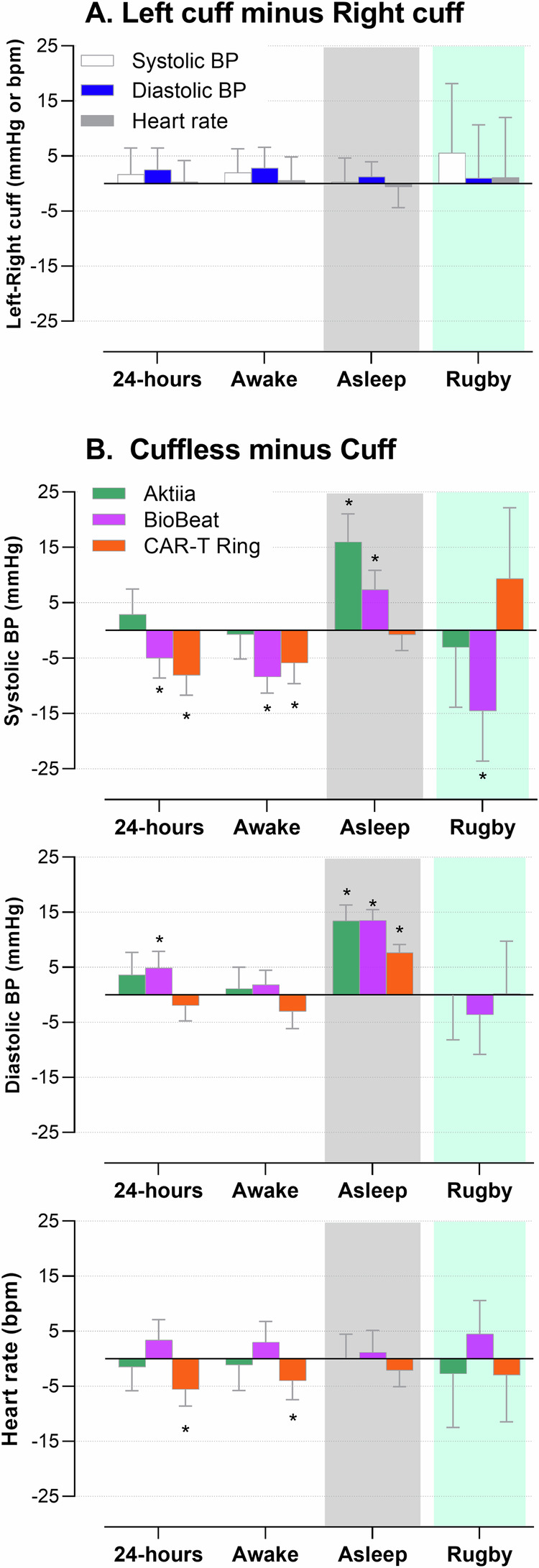
Differences in average blood pressure and heart rate over 24 h using various blood pressure devices. **A** Differences between left and right arm cuff-based devices, and differences between cuffless and cuff-based devices for **B** systolic blood pressure, diastolic blood pressure, and heart rate.

It is clear from the figures that each device type has its own features that may require highlighting.

- Where the cuff-based ambulatory devices were programmed to take readings every 20 min, the cuffless devices are usually programmed to only take a reading when the person is not moving. How would this influence ‘true’ blood pressure? Since it is known that a cuff inflation causes an anxiety response [12], would that mean that cuff-based ambulatory blood pressure should be higher overall than cuffless measurements?
- Also, although the data presented here were generated from current cuffless devices, the companies continuously strive to improve software algorithms, and therefore often launch updates of their mobile device applications. Hence, improved versions may already be available for some.
- Where a patient can usually only endure one 24-h cuff-based blood pressure measurement at a time, cuffless devices are used for weeks and months without the user being aware that readings are taken. The benefit of weeks or months’ worth of data compared to a single 24-h period, is perhaps underestimated.
- The number of readings taken by the different devices also varies substantially. For instance, during nighttime 16 cuff blood pressure readings were taken, 10 Aktiia readings, 10 Biobeat readings, and 58 CAR-T readings.

With blood pressure changing all the time, it is likely that when more readings are taken, it may be better than fewer. In fact, when reflecting on current clinical practice mostly relying on a single snapshot clinic blood pressure taken (often imprecisely) for decision-making [22], one cannot help but wondering whether it is time to completely overhaul how blood pressure is measured in primary care. Current blood pressure measurement practices across thousands of busy clinics is certainly not ‘good enough’—the question is when cuffless readings will be good enough?
